# Performance of Dried Blood Spot Cards for Serologic Detection of HPV16 Antibodies in Oropharyngeal Squamous Cell Carcinoma Patients

**DOI:** 10.3390/microorganisms13112558

**Published:** 2025-11-10

**Authors:** Maisha Maliha Rahman, Soma Bose, Li Chen, Jessica L. Burris, Rony Aouad, Susanne M. Arnold, Melvyn Yeoh, Birgitta Michels, Tim Waterboer, Krystle A. Lang Kuhs

**Affiliations:** 1 Department of Epidemiology and Environmental Health, College of Public Health, University of Kentucky, Lexington, KY 40536, USA; maisha.rahman@uky.edu (M.M.R.); soma.bose@uky.edu (S.B.); 2 Division of Cancer Biostatistics, College of Medicine, University of Kentucky, Lexington, KY 40536, USA; lichenuky@uky.edu; 3 Markey Cancer Center, University of Kentucky, Lexington, KY 40536, USA; burris.jessica@gmail.com (J.L.B.); rony.aouad@uky.edu (R.A.); susanne.arnold@uky.edu (S.M.A.); melvyn.yeoh@uky.edu (M.Y.); 4 Department of Psychology, College of Arts and Sciences, University of Kentucky, Lexington, KY 40536, USA; 5 Department of Internal Medicine, College of Medicine, University of Kentucky, Lexington, KY 40536, USA; 6 Division of Oral and Maxillofacial Surgery, College of Dentistry, University of Kentucky, Lexington, KY 40536, USA; 7 Immunology, Infection & Cancer Research Program, Division of Infections and Cancer Epidemiology, German Cancer Research Center (DKFZ), 69120 Heidelberg, Germany; b.michels@dkfz-heidelberg.de (B.M.); t.waterboer@dkfz-heidelberg.de (T.W.)

**Keywords:** human papillomavirus, HPV, antibodies, oropharyngeal squamous cell carcinoma, OPSCC, dried blood spot cards, DBS, HPV16 E6

## Abstract

Antibodies against the human papillomavirus type 16 (HPV16) E6 oncoprotein are promising biomarkers for the early detection of HPV-driven oropharyngeal squamous cell carcinoma (HPV+OPSCC). Standard serologic testing is challenging in underserved regions with high HPV+OPSCC incidence. Dried blood spot (DBS) cards offer a low-resource alternative but remain unevaluated for HPV antibody detection. A total of 25 OPSCC patients who provided paired serum (venipuncture) and DBS (finger-prick) samples were recruited from the University of Kentucky. HPV16 antibodies (L1, E1, E2, E4, E6, E7) were measured using multiplex serology, quantified as median fluorescence intensity (MFI) and dichotomized using established cutoffs. Correlation between serum and DBS MFI values was evaluated using linear regression and Bland–Altman plots, whereas sensitivity, specificity, and Cohen’s kappa assessed agreement. Mean MFI levels were lower in DBS than serum but were strongly correlated (R = 0.73 to 0.96; HPV16 E6 = 0.83). For HPV16 E6, DBS sensitivity was 90% (95% CI: 68–99) and specificity 100% (95% CI: 48–100), with kappa = 0.787. Specificity was 100% across all markers, while sensitivity varied from 0% (L1) to 100% (E2). DBS cards are accurate, inexpensive, and a scalable alternative for HPV16 E6 antibody detection, particularly in medically underserved regions, though further validation is needed.

## 1. Introduction

In recent years, the incidence of oropharyngeal squamous cell carcinoma (OPSCC)—a subtype of head and neck cancer—has risen sharply across many developed countries [1,2,3,4,5,6,7,8]. This rise in incidence is attributed to an increase in HPV-associated OPSCC (HPV+OPSCC) [7]. It is estimated that HPV infection currently accounts for 80% of OPSCC cases diagnosed in the United States [9]. HPV type 16 (HPV16) is the predominant type, accounting for 80 to 90% of HPV+OPSCCs [10,11,12]. In contrast, the second-most prevalent types, HPV18 and HPV33, account for less than 10% of OPSCC cases [11]. A total of 80% of all HPV-OPSCC cases occur among males and non-Hispanic white individuals [13]. Because of the lack of identifiable pre-malignant lesions for HPV-related head and neck cancers, there are currently no effective screening methods for early detection of HPV+OPSCC [14].

A blood-based biomarker, antibodies against the HPV16 E6 oncoprotein, are present in approximately 90% of HPV+OPSCC patients and appear more than a decade before diagnosis [15,16,17]. As HPV16 E6 seropositivity is not associated with any other head and neck cancer sites and several cases of HPV+OPSCC have been diagnosed among asymptomatic individuals with HPV16 E6 seropositivity, it may be a viable tool for early detection, enabling targeted screening efforts [15,16].

However, screening for HPV16 E6 seropositivity can pose logistical challenges, particularly in medically underserved areas. Standard antibody testing typically requires venipuncture, which necessitates trained personnel, specific equipment and strict handling protocols—including a cold chain for sample preservation. In contrast, dried blood spot (DBS) cards are a practical alternative to standard antibody testing with venipuncture. DBS cards offer the advantage of self-collection, require no special processing or equipment prior to storage, and are stable without refrigeration. Emerging evidence suggests that they can serve as a promising alternative to venipuncture for SARS-CoV-2 serolomics, COVID-19 antibody testing, detecting Measles, Rubella, Midazolam and STI testing [18,19,20,21,22]. In this proof-of-concept study, we examine the validity of using DBS cards to evaluate HPV antibodies using multiplex serology.

## 2. Materials and Methods

### 2.1. Study Population

Patients undergoing treatment or receiving post-treatment medical care for OPSCC were recruited from the Markey Cancer Center’s Head, Neck and Respiratory Clinic at the University of Kentucky (Lexington, KY, USA) between 7 May 2022 to 27 April 2023. Inclusion criteria for the study were an age of ≥21 years and an OPSCC diagnosis. Eligible patients were identified via review of electronic health records. Patients were invited to participate during a regularly scheduled visit where written informed consent was obtained on iPads using a HIPAA compliant REDCap database (REDCap 15.8.2 © 2025 Vanderbilt University). At the time of enrollment, participants were asked to provide two blood samples; one collected through venipuncture by a clinic phlebotomist, and another collected with a finger prick and DBS card by the research coordinator. To reduce the number of procedures, efforts were made to collect the research’s venous blood draw during a patient’s routine lab visit, and for a subset of participants (3/25), previously banked research blood samples that were collected as part of the ORIEN Total Cancer Care Network were used [23]. Forty-seven participants were enrolled over an almost 1-year period; 25 had both a DBS card and serum sample available for serologic analyses and were included in this study.

### 2.2. Sample Collection

DBS cards were collected via finger prick using a 2 mm × 1.5 mm lancet (BD Microtainer Becton, Dickinson and Company, Dublin, Ireland) on Whatman 903 protein saver blood collection cards (Whatman, Maidstone, UK) and processed per manufacturer specifications. Once dry, all cards were stored at 4 °C, individually packaged in gas-impermeable zipper bags containing a desiccant packet and a humidity indicator card. Serum from whole blood was processed as follows: blood was drawn by venipuncture in 2 × 5 mL serum separator collection tubes (SSTs), blood was allowed to clot for 30 min, the tubes were centrifuged at 2000× *g* for 10 min at room temperature, and 0.5 mL aliquots of the supernatant (serum) were transferred into cryogenic tubes (Nunc #375353, Roskilde, Denmark) for storage at −80 °C.

### 2.3. HPV Multiplex Serology

Although HPV16 E6 serology was our main outcome of interest, we also tested for seroreactivity to five other HPV16 proteins: E1, E2, E4, E7 and L1. De-identified DBS cards and serum samples were shipped at ambient temperature (DBS cards) or dry-ice packaging (serum) to the German Cancer Research Center (DKFZ) for multiplex serology testing. The DBS cards were stored at 4 °C until testing. To ensure comparable antibody concentrations from the DBS cards and the serum samples, established protocols were used. In short, antibodies from each DBS card were eluted from a DBS punch followed by overnight agitation in PBS at 4 °C. Seroreactivity against the HPV16 proteins was determined using multiplex serology, an antibody detection method based on a glutathione S-transferase (GST) capture ELISA in combination with fluorescent bead technology [24,25]. In brief, each antigen of interest is bacterially expressed as double fusion protein containing a C-terminal undecapeptide tag from the large T simian virus 40 antigen and an N-terminal GST-tag. Antigens are immobilized on a specific bead set of glutathione–casein-coated polystyrene beads. Each bead set is characterized by a spectrally distinct fluorophore (SeroMAP^TM^ Microspheres, Luminex Corp./Diasorin, Austin, TX, USA). Serum samples or DBS eluates were incubated with the pooled bead sets loaded with the respective antigens. Successful binding of blood antibodies to the bead-bound antigens is detected by incubation with a biotinylated anti-human antibody and subsequent streptavidin–R phycoerythrin reaction. Eventually, a Luminex 200 (Luminex Crop./Diasorin, Austin, TX, USA) device was used to simultaneously discriminate between bead sets by internal color and to quantify the signal from antibodies bound to the respective antigen, reported in Median Fluorescence Intensity (MFI). For each bead sort, at least 100 beads were measured.

The HPV multiplex assay used in this study is currently the most widely used and validated method for detecting HPV-specific antibodies in OPSCC. This assay is highly reproducible; an analysis of serum samples from 114 OPC patients found an intra-individual correlation of 1.0 for HPV16 E6 seropositivity [15]. Successful binding of the double fusion antigen peptides to the beads was verified via monoclonal antibody binding to the C-terminal tag-end. Sample background binding was determined by measuring signals from a bead set that was loaded with GST-tag only. Background binding of reagents to the beads and autofluorescence were determined by incubating beads without human serum/DBS eluate and subsequent signal measurements. Background measurements from wells without sample material and GST-background were subtracted from the values measured for each sample. Internal positive and negative controls were included on each plate and reaction levels were compared to prior experiments. Antibody levels were quantified as median fluorescence intensity (MFI) and dichotomized (positive/negative) based on the following cutpoints: E6—484, E1—200; E2—679, E4—876, E5—548, L1—422 [26]. The dichotomized data for each participant can be found in Supplementary Table S1.

### 2.4. Statistical Analysis

Demographic and clinical characteristics of the study participants were evaluated. Due to the low counts in two categories of the race variable (Black or African American, and More than one race), they have been collapsed to one category named “Other”. Individual log_10_ (MFI) values for serum or DBS for each patient were plotted on a scatter plot to visualize the association between the two measurements. Correlation between sample sets was evaluated using linear regressions between the log_10_ (MFI) values for serum and DBS for each oncoprotein. The log_10_ of mean MFI was plotted against the ratio of MFI (serum/DBS) on a Bland–Altman plot for each patient to observe any trends. For each HPV16 protein evaluated, sensitivity and specificity were calculated with DBS using the serum antibody results as the gold standard. Sensitivity was calculated as the proportion of seropositive serum samples that tested seropositive with DBS. Specificity was calculated as the proportion of seronegative serum samples that tested seronegative with DBS. Exact Binomial Confidence Intervals (Clopper–Pearson) were calculated for each sensitivity and specificity. These were then used to calculate the Cohen’s Kappa statistic [27]. All the analyses have been conducted using R statistical software: version 4.4.2 [28].

## 3. Results

### 3.1. Participant Characteristics

The median age of the study participants was 63 (interquartile range [IQR]: 58–67). The majority were male, white, and had a history of cigarette smoking. Most participants had finished treatment (68%) at the time of enrollment, 28% were still undergoing treatment and 4% were in pre-treatment status (Table 1).

### 3.2. Comparison of MFI Antibody Levels in DBS and Serum

**Figure 1:**
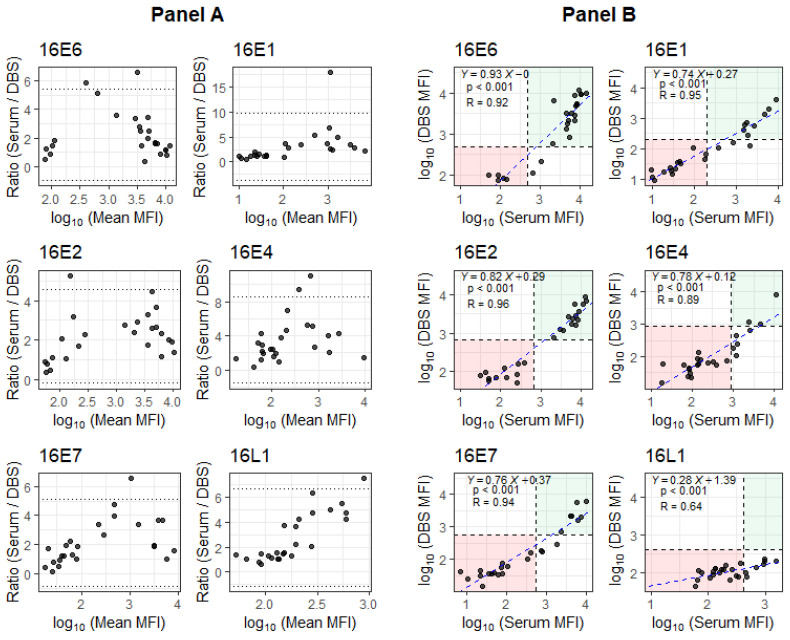
Evaluation of DBS performance relative to serum antibody levels for OPSCC patients. **Panel A**: Bland–Altman plots showing the ratio between paired antibody responses across the log_10_ of mean MFI scale. Dotted lines are located at the mean ± 1.96 standard deviation of ratios. **Panel B**: Scatter plots of log_10_ (MFI) between serum and DBS, where the blue dashed lines represent the fitted linear regressions and the black dashed lines represent cutoff values for seropositivity. The linear model shows the trend between the log_10_(MFI) values for serum and DBS (with Y = log_10_(DBS[MFI]), X = log_10_(Serum[MFI]), *p*-value for slope and R for the model).

While the mean MFI antibody levels were lower for DBS compared to serum (Figure 1: Panel A), MFIs for DBS and serum were strongly correlated (Figure 1: Panel B). The R for HPV16 E6 antibodies was 0.92. For HPV16 E2 antibodies, another strong marker of HPV+OPSCC, the R was 0.96. The R values for the rest of the oncoproteins ranged from 0.64 to 0.95, with HPV16 L1 having the weakest correlation (R = 0.64).

### 3.3. Concordance of DBS with Serum for HPV16 Seropositivity

For each HPV16 protein evaluated, MFI levels were dichotomized as seropositive or seronegative to determine the agreement between serum and DBS; serum results were used as the gold standard (Table 2). Among the 20 HPV16 E6 seropositive participants (serum), 18 were also seropositive on DBS (Sensitivity: 90% [95% CI: 68 to 99]); all five participants who tested HPV16 E6 seronegative on serum also tested seronegative on DBS (Specificity: 100% [95% CI: 48 to 100]; kappa: 0.787). For HPV16 E2, there was a perfect agreement between serum and DBS results (Sensitivity: 100% [95% CI: 77 to 100], Specificity: 100% [95% CI: 72 to 100], kappa: 1.00). For the remaining antibodies, specificity was 100% for all markers evaluated, while sensitivity ranged from 0% (HPV16 L1) to 73% (HPV16 E1).

## 4. Discussion

This study is the first to assess the accuracy of DBS for detecting antibodies against HPV16. When compared to serum-based antibody testing, which served as the reference standard, DBS yielded lower antibody concentrations but demonstrated strong concordance, particularly for HPV16 E6 and E2 antibodies, both of which are well-established indicators of HPV+OPSCC risk. Although DBS has been used for antibody detection since the 1860s [29], our findings highlight its potential as a cost-effective, convenient and reliable alternative to traditional serum collection for identifying HPV16 E6 seropositive individuals.

Although this is the first study to assess the use of dried blood spots (DBS) for HPV antibody detection, our findings align with previous research comparing DBS and venipuncture for identifying viral antibodies [18,19,20,21,22]. For example, one study involving 142 participants reported lower antibody concentrations from DBS compared to venipuncture, yet still demonstrated a strong correlation between the two methods for SARS-CoV-2 seropositivity [18]. Another study comparing DBS and venipuncture for SARS-CoV-2 anti-spike IgG antibodies reported a Cohen’s Kappa statistic of 0.91 when DBS samples were collected by study nurses and a perfect agreement (Kappa = 1.0) when collected by participants themselves [19]. DBS has also been found to be an excellent alternative to venipuncture for measles and rubella antibody testing [20].

This study has strengths and some limitations. A primary limitation was the small sample size of 25 participants. However, a key strength of our study lies in the within-subject design—both DBS and venipuncture samples were collected from each participant [30]. Our study also focused on OPSCC patients, which maximized our ability to compare the two methods, given the high prevalence of HPV antibodies in this population [31]. Another limitation of this study is that HPV16 E6 antibody quantification was performed using a single serologic assay. To our knowledge, no other available method demonstrates decent sensitivity and specificity for detecting HPV16 E6 antibodies in blood-based samples. Additionally, our serologic analysis focused on samples with high antibody titers. Comparing DBS with venipuncture in low-concentration serum samples was beyond the scope of this study and represents a clear limitation. All samples were analyzed using the gold-standard multiplex serology assay for HPV antibodies, ensuring consistency and accuracy in measurement. While most participants provided DBS and venipuncture samples at the same study visit, a subset of participants had these samples collected asynchronously. Nevertheless, this is unlikely to affect our study results as our previous study found that HPV16 E6 antibodies remain stable years after diagnosis and successful treatment [16]. It should be noted that antibodies directed against HPV16 proteins may cross-react with other closely related α-papillomavirus types, such as HPV18, due to shared antigenic epitopes [32]. Although our study focused on HPV16-driven OPSCC, which accounts for the vast majority of HPV-related cases, a small degree of cross-reactivity may not be excluded. Nevertheless, given that HPV16 is responsible for approximately 90% of HPV-positive OPSCC, this limitation is unlikely to substantially affect the interpretation of our results or their relevance to HPV-driven disease screening [10,11]. Finally, while we found a strong agreement for the two HPV16 antibodies most strongly associated with OPSCC risk, HPV16 E6 and HPV16 E2, we noted a poor agreement for the main marker of HPV16 exposure/immunity, L1 antibodies [15]. These findings suggest that DBS may be a reliable method in studies focused on OPSCC risk through HPV16 E6 and E2 antibody testing; however, DBS may be less suitable for studies focused on HPV16 exposure or immunity.

Overall, our findings indicate that DBS cards offer a cost-effective, efficient and accurate alternative to venipuncture for screening for HPV16 E6 antibodies. This method is particularly advantageous for large-scale screening studies where thousands of participants need to be screened to find the 0.5% to 0.8% of participants harboring HPV16 E6 antibodies [17,33]. Despite these promising results, the relatively small sample size underscores the need for larger studies to confirm and validate the results.

## Figures and Tables

**Table 1:** Demographic characteristics of study participants at the time of enrollment.

Characteristic	N = 25 ^1^
Age	63.00 (58.00, 67.00)
Sex at Birth	
Male	23 (92%)
Female	2 (8%)
Race	
White	23 (92%)
Other ^2^	2 (8%)
Treatment Status	
Pre-treatment	1 (4%)
Currently Undergoing Treatment	7 (28%)
Finished Treatment	17 (68%)
Stage of Cancer	
I/II	8 (32%)
III/IV	13 (52%)
Incomplete	1 (4%)
Unstaged	3 (12%)
P16 Status ^3^	
Positive	19 (95%)
Negative	1 (5%)
N. Missing Observations	5
Smoking Status	
Never	9 (36%)
Current	5 (20%)
Former	11 (44%)
Cigarette Pack Years	27.50 (13.50, 40.00)
N. Missing Observations	9

^1^ Median (Q1, Q3); N (%); ^2^ Other contains “Black or African American” and “More than one race”. ^3^ P16 is the gold-standard clinical method for assessing HPV status. Tumors testing p16 positive are assumed to be HPV+OPSCC.

**Table 2:** Sensitivity and specificity table for HPV16 antibodies.

Marker Names	No. Positives with Serum	No. Positives with DBS	Sensitivity % [95% CI]	No. Negatives with Serum	No. Negatives with DBS	Specificity % [95% CI]	Cohen’s Kappa
16E6	20	18	90% [68%, 99%]	5	5	100% [48%, 100%]	0.787
16E1	11	8	72.7% [39%, 94%]	14	14	100% [77%, 100%]	0.786
16E2	14	14	100% [77%, 100%]	11	11	100% [72%, 100%]	1.000
16E4	8	3	37.5% [9%, 76%]	17	17	100% [80%, 100%]	0.645
16E7	10	7	70% [35%, 93%]	15	15	100% [78%, 100%]	0.781
16L1	7	0	0% [0%, 41%]	18	18	100% [81%, 100%]	0.529

## Data Availability

The data presented in this study are available on request from the corresponding author due to privacy and ethical restrictions involving patient information.

## Supplementary Materials

The following supporting information can be downloaded at: https://www.mdpi.com/article/10.3390/microorganisms13112558/s1, Table S1: Dichotomized sero-reactivity for each biomarker for each participant.

## Abbreviations

The following abbreviations are used in this manuscript:

HPV16	Human papillomavirus type 16
HPV+OPSCC	HPV-driven oropharyngeal squamous cell carcinoma
OPSCC	Oropharyngeal Squamous Cell Carcinoma
DBS	Dried Blood Spots
MFI	Median Fluorescence Intensity

## References

[1] Hocking J.S., Stein A., Conway E.L., Regan D., Grulich A., Law M., Brotherton J.M.L. (2011). Head and neck cancer in Australia between 1982 and 2005 show increasing incidence of potentially HPV-associated oropharyngeal cancers. Br. J. Cancer.

[2] Blomberg M., Nielsen A., Munk C., Kjaer S.K. (2011). Trends in head and neck cancer incidence in Denmark, 1978–2007: Focus on human papillomavirus associated sites. Int. J. Cancer.

[3] Reddy V., Cundall-Curry D., Bridger M. (2010). Trends in the incidence rates of tonsil and base of tongue cancer in England, 1985–2006. Ann. R. Coll. Surg. Engl..

[4] Syrjänen S. (2004). HPV infections and tonsillar carcinoma. J. Clin. Pathol..

[5] Ioka A. (2005). Trends in Head and Neck Cancer Incidence in Japan during 1965–1999. Jpn. J. Clin. Oncol..

[6] Braakhuis B.J.M., Visser O., René Leemans C. (2009). Oral and oropharyngeal cancer in The Netherlands between 1989 and 2006: Increasing incidence, but not in young adults. Oral Oncol..

[7] Chaturvedi A.K., Engels E.A., Anderson W.F., Gillison M.L. (2008). Incidence Trends for Human Papillomavirus–Related and –Unrelated Oral Squamous Cell Carcinomas in the United States. J. Clin. Oncol..

[8] Gillison M.L., Alemany L., Snijders P.J.F., Chaturvedi A., Steinberg B.M., Schwartz S., Castellsagué X. (2012). Human Papillomavirus and Diseases of the Upper Airway: Head and Neck Cancer and Respiratory Papillomatosis. Vaccine.

[9] Gribb J.P., Wheelock J.H., Park E.S. (2023). Human Papilloma Virus (HPV) and the Current State of Oropharyngeal Cancer Prevention and Treatment. Del. J. Public Health.

[10] Schache A.G., Powell N.G., Cuschieri K.S., Robinson M., Leary S., Mehanna H., Rapozo D., Long A., Cubie H., Junor E. (2016). HPV-Related Oropharynx Cancer in the United Kingdom: An Evolution in the Understanding of Disease Etiology. Cancer Res..

[11] Kreimer A.R., Clifford G.M., Boyle P., Franceschi S. (2005). Human Papillomavirus Types in Head and Neck Squamous Cell Carcinomas Worldwide: A Systematic Review. Cancer Epidemiol. Biomark. Prev..

[12] France World Health Organization (2007). IARC Monographs on the Evaluation of Carcinogenic Risks to Humans Human Papillomaviruses.

[13] Damgacioglu H., Sonawane K., Zhu Y., Li R., Balasubramanian B.A., Lairson D.R., Giuliano A.R., Deshmukh A.A. (2022). Oropharyngeal Cancer Incidence and Mortality Trends in All 50 States in the US, 2001–2017. JAMA Otolaryngol. Head Neck Surg..

[14] Galati L., Chiocca S., Duca D., Tagliabue M., Simoens C., Gheit T., Arbyn M., Tommasino M. (2022). HPV and head and neck cancers: Towards early diagnosis and prevention. Tumour Virus Res..

[15] Kreimer A.R., Johansson M., Waterboer T., Kaaks R., Chang-Claude J., Drogen D., Tjønneland A., Overvad K., Quirós J.R., González C.A. (2013). Evaluation of Human Papillomavirus Antibodies and Risk of Subsequent Head and Neck Cancer. J. Clin. Oncol..

[16] Lang Kuhs K.A., Kreimer A.R., Trivedi S., Holzinger D., Pawlita M., Pfeiffer R.M., Gibson S.P., Schmitt N.C., Hildesheim A., Waterboer T. (2017). Human papillomavirus 16 E6 antibodies are sensitive for human papillomavirus–driven oropharyngeal cancer and are associated with recurrence. Cancer.

[17] Kreimer A.R., Johansson M., Yanik E.L., Katki H.A., Check D.P., Lang Kuhs K.A., Willhauck-Fleckenstein M., Holzinger D., Hildesheim A., Pfeiffer R. (2017). Kinetics of the Human Papillomavirus Type 16 E6 Antibody Response Prior to Oropharyngeal Cancer. JNCI J. Natl. Cancer Inst..

[18] Jeske R., Merle U., Müller B., Waterboer T., Butt J. (2022). Performance of Dried Blood Spot Samples in SARS-CoV-2 Serolomics. Microorganisms.

[19] Sims M.D., Podolsky R.H., Childers K.L., Higgins B., Trueman J., Homayouni R., Voss D.R., Berkiw-Scenna N., Keil H., Kennedy R.H. (2023). Dried blood spots are a valid alternative to venipuncture for COVID-19 antibody testing. J. Immunol. Methods.

[20] Pinsky N.A., Loepfe T.R., Jacobson R.M., Vierkant R.A., Poland G.A. (2003). Comparison of Fingerstick Versus Venipuncture for Antibody Testing of Measles and Rubella. Scand. J. Infect. Dis..

[21] Lachance S., Théberge M.-C., Havard G., Anctil D., Barrière O., Croteau S., Pellerin B., Lévesque A. (2016). Comparison of Blood Microsampling with DBS and Conventional Blood Collection Techniques Used in A Midazolam Biostudy. Bioanalysis.

[22] Nieuwenburg S.A., Bruisten S.M., Heijman T., Vermeulen W., Van Dam A.P., Schim Van Der Loeff M.F., De Vries H.J.C. (2024). Use of Home-Based Self-Collected Dried Blood Spots to Test for Syphilis, Human Immunodeficiency Virus, Hepatitis C and B Virus Infections and Measuring Creatinine Concentration. Sex. Transm. Dis..

[23] Fenstermacher D.A., Wenham R.M., Rollison D.E., Dalton W.S. (2011). Implementing Personalized Medicine in a Cancer Center. Cancer J..

[24] Waterboer T., Sehr P., Pawlita M. (2006). Suppression of non-specific binding in serological Luminex assays. J. Immunol. Methods.

[25] Sitas F., Egger S., Urban M.I., Taylor P.R., Abnet C.C., Boffetta P., O’Connell D.L., Whiteman D.C., Brennan P., Malekzadeh R. (2012). InterSCOPE Study: Associations Between Esophageal Squamous Cell Carcinoma and Human Papillomavirus Serological Markers. JNCI J. Natl. Cancer Inst..

[26] Lang Kuhs K.A., Anantharaman D., Waterboer T., Johansson M., Brennan P., Michel A., Willhauck-Fleckenstein M., Purdue M.P., Holcátová I., Ahrens W. (2015). Human Papillomavirus 16 E6 Antibodies in Individuals without Diagnosed Cancer: A Pooled Analysis. Cancer Epidemiol. Biomark. Prev..

[27] McHugh M.L. (2012). Interrater reliability: The kappa statistic. Biochem. Medica.

[28] R Foundation for Statistical Computing (2021). R: A Language and Environment for Statistical Computing.

[29] Lim M.D. (2018). Dried Blood Spots for Global Health Diagnostics and Surveillance: Opportunities and Challenges. Am. J. Trop. Med. Hyg..

[30] Charness G., Gneezy U., Kuhn M.A. (2012). Experimental methods: Between-subject and within-subject design. J. Econ. Behav. Organ..

[31] Kreimer A.R., Brennan P., Lang Kuhs K.A., Waterboer T., Clifford G., Franceschi S., Michel A., Willhauck-Fleckenstein M., Riboli E., Castellsagué X. (2015). Human Papillomavirus Antibodies and Future Risk of Anogenital Cancer: A Nested Case-Control Study in the European Prospective Investigation into Cancer and Nutrition Study. J. Clin. Oncol..

[32] Sehr P., Müller M., Höpfl R., Widschwendter A., Pawlita M. (2002). HPV antibody detection by ELISA with capsid protein L1 fused to glutathione S-transferase. J. Virol. Methods.

[33] Brenner N., Mentzer A.J., Hill M., Almond R., Allen N., Pawlita M., Waterboer T. (2020). Characterization of human papillomavirus (HPV) 16 E6 seropositive individuals without HPV-associated malignancies after 10 years of follow-up in the UK Biobank. eBioMedicine.

