# Supplementary material for: Performance of Dried Blood Spot Cards for Serologic Detection of HPV16 Antibodies in Oropharyngeal Squamous Cell Carcinoma Patients
Source: Microorganisms. 2025 Nov 10;13(11):2558. doi: 10.3390/microorganisms13112558 (PMC12654639; doi:10.3390/microorganisms13112558)
Supplement: Supplementary file 1 [file microorganisms-13-02558-s001.zip › microorganisms-3871813-supplementary.pdf]

microorganisms

MDPI

Supplementary Table S1: Dichotomized sero-reactivity for each biomarker for each participant

	**DBS**						**Serum**					
	**HPV16E1**	**HPV16E2**	**HPV16E4**	**HPV16E6**	**HPV16E7**	**HPV16L1**	**HPV16E1**	**HPV16E2**	**HPV16E4**	**HPV16E6**	**HPV16E7**	**HPV16L1**
**Cutoffs**	200	679	876	484	548	422	200	679	876	484	548	422
**ID**												
**1**	0	0	0	1	0	0	0	0	0	1	1	0
**2**	1	1	0	1	0	0	1	1	0	1	1	0
**3**	0	0	0	0	0	0	0	0	0	0	0	0
**4**	0	0	0	0	0	0	0	0	0	0	0	1
**5**	0	1	0	1	0	0	0	1	1	1	0	0
**6**	0	1	0	1	1	0	1	1	1	1	1	1
**7**	0	1	0	1	1	0	1	1	0	1	1	0
**8**	1	1	0	1	0	0	1	1	0	1	0	0
**9**	0	1	0	1	1	0	0	1	1	1	1	0
**10**	1	1	0	1	1	0	1	1	1	1	1	0
**11**	1	1	0	1	0	0	1	1	0	1	0	0
**12**	0	1	0	1	1	0	0	1	0	1	1	0
**13**	0	0	0	0	0	0	0	0	0	0	0	0
**14**	0	0	0	0	0	0	0	0	0	1	0	1
**15**	0	0	0	0	0	0	0	0	0	1	0	1
**16**	1	1	1	1	0	0	1	1	1	1	0	0

**17**	1	1	0	1	1	0	1	1	0	1	1	1
**18**	0	0	0	0	0	0	0	0	0	0	0	1
**19**	0	0	1	1	0	0	1	0	1	1	0	0
**20**	1	0	0	1	1	0	1	0	0	1	1	0
**21**	0	0	0	0	0	0	0	0	0	0	0	0
**22**	1	1	1	1	0	0	1	1	1	1	1	1
**23**	0	1	0	1	0	0	0	1	0	1	0	0
**24**	0	1	0	1	0	0	0	1	1	1	0	0
**25**	0	0	0	1	0	0	0	0	0	1	0	0
